# Identification of subgroups of early-stage mycosis fungoides patients with increased itch and impaired quality of life

**DOI:** 10.3389/fonc.2025.1524353

**Published:** 2025-02-28

**Authors:** Julia Nenonen, Anna H. Winther, Pontus Jonsson, Lina U. Ivert, Hanna Brauner

**Affiliations:** ^1^ Division of Dermato-Venerology, Department of Medicine, Solna, and Center for Molecular Medicine, Karolinska Institutet, Stockholm, Sweden; ^2^ Department of Dermatology, Karolinska University Hospital, Stockholm, Sweden

**Keywords:** quality of life, mycosis fungoides, cutaneous T cell lymphoma, depression, itch

## Abstract

**Introduction:**

Mycosis fungoides (MF), Sézary syndrome (SS) and other cutaneous T-cell lymphomas (CTCLs) can have a severe impact on quality of life (QoL) and itch, but early MF is insufficiently investigated despite representing most patients. This single center study investigated associations between QoL/itch/depressive symptoms and clinical phenotypes in patients with CTCL with particular focus on early MF-stages.

**Methods:**

Patients were included during routine dermatological care. The primary outcomes included Dermatology Life Quality Index (DLQI), EuroQoL 5D (EQ-5D) index, Montgomery-Åsberg Depression Rating Scale – Self report (MADRS-S), and itch measured with a visual analogue scale (VAS-itch).

**Results:**

In the total CTCL cohort (n=76), median EQ-5D index was impaired in female vs male patients (0.73 vs 0.85, p = 0.040). Among early MF patients (n=58), increased disease activity correlated with impaired DLQI (r = 0.413, p = 0.0014) and EQ-5D index (r = -0.317, p = 0.0161). Early MF patients with plaques vs only patches reported impaired EQ-5D index (median 0.725 vs 0.848, p = 0.0032) and increased itch (median VAS 3.27 vs 0.43, p = 0.0006). MF patients with stage IB vs IA reported impaired DLQI (median 5.00 vs 1.00, p = 0.0006), impaired EQ-5D index (median 0.725 vs 0.848, p = 0.0040) and increased itch (median VAS 3.37 vs 0.54, p = 0.0487).

**Discussion:**

Although early MF patients reported generally a mild impact on QoL, this study highlights the need for disease management optimization for subgroups of early MF patients, including those with plaques, stage IB and higher disease activity.

## 1 Introduction

Cutaneous T cell lymphoma (CTCL) is a heterogeneous group of malignancies with variable clinical presentation and prognosis. The most common CTCL subtype, mycosis fungoides (MF), accounts for nearly 60% of all CTCL cases (1). Other subtypes include Sézary syndrome (SS), lymphomatoid papulosis (LyP) and primary cutaneous anaplastic large cell lymphoma (PC-ALCL). Approximately 70% of MF patients are diagnosed at early clinical stages IA–IIA, characterized by patch and/or plaque lesions of the skin and no or limited systemic spread of the disease (2). The prognosis for early-stage MF is generally good with a 10-year overall survival of 52–88%, but 16% of the patients progress to advanced-stage disease within five years of diagnosis (3, 4). As there are no curative treatments for early-stage MF, the aim of the available therapies is to prevent disease progression, control symptoms, and promote high quality of life (QoL).

Given the generally slow clinical course, frequently accompanied with chronic pruritus, pain, and other distressing symptoms, CTCL can have a severe impact on QoL (5). MF, including the early-stage disease, has been associated with lower QoL compared with several other chronic diseases such as diabetes, kidney disease treated with hemodialysis, and cancer (6). Beyond the physical aspects of QoL, CTCL patients can have disruptions in emotional and social wellbeing (5). Only few studies have explored the association between CTCL and depression and with conflicting results (7, 8). Factors associated with worse QoL in CTCL patients include female sex, alopecia, and pruritus (9).

As a central aim of CTCL management is to improve QoL, it is essential to investigate factors possibly associated with poor QoL in CTCL patients, particularly in early-stage MF, for which no curative treatment exists. A systematic review of QoL in different CTCL stages suggests that advanced-stage MF/SS has a greater impact on QoL than early-stage disease (10). More studies on QoL in the early stages of MF are needed to identify subgroups of patients in need of intensified support, including distinguishing early stages IA vs IB and other clinical phenotypes. For example, analysis of the effect of thinner patch lesions vs thicker plaque lesions on QoL is lacking. Furthermore, the affected body surface area (BSA) and the types of lesions vary within early stages in patients with MF, with stage IB, affecting anywhere between 10% and 79% of BSA. Therefore, analysis of disease activity in MF/SS, as measured with the modified Severity-Weighted Assessment Tool (mSWAT) (11), can generate a higher resolution. Previous studies on a possible relationship between QoL and mSWAT have been based on small cohorts or only newly diagnosed CTCL patients and have failed to find independent associations between disease activity and QoL variables (9, 12).

This study aimed to describe QoL, itch, and depressive symptoms in patients with CTCL in routine dermatological care, with particular focus on early MF (stages IA–IIA). Another aim was to investigate possible associations between worse QoL/itch/depressive symptoms and early MF lesion type, stage as well as disease activity.

## 2 Patients and methods

### 2.1 Study design

This cross-sectional study included CTCL patients from the Department of Dermatology, Karolinska University Hospital, Stockholm, Sweden. Patients were included in the study during regular clinical visits at the clinic between 15 October 2019 and 23 January 2024. Both newly diagnosed patients and patients with an established diagnosis were included in the study. The inclusion criteria were as follows: (i) age >18 years, with ability to comprehend and complete questionnaires, and (ii) histopathologically and clinically confirmed CTCL diagnosis.

### 2.2 Outcome measures

The primary outcome measures included patient-reported data from the following questionnaires: Dermatology Life Quality Index (DLQI) (13), EuroQoL 5D (EQ-5D) (14), Montgomery-Åsberg Depression Rating Scale – Self report (MADRS-S) (15), and a visual analogue scale for itch (VAS-itch) (16). The term quality of life, “QoL”, is used throughout the study for data from DLQI and/or EQ-5D and the term “QoL measures” is used for data from all four questionnaires. The total DLQI score is based on 10 items that can be grouped into six subscales, assessing “symptoms and feelings” (items 1-2), “daily activities” (items 3-4), “leisure” (items 5-6), “work and school” (item 7), “personal relationships” (items 8-9), and “treatment” (item 10). The total DLQI score ranges from 0 to 30, with higher scores indicating worse QoL. For DLQI, a minimal clinically important difference (MCID) of 4.0 points has been suggested in patients with inflammatory skin disease (17). We applied the same MCID in interpreting our results, as MF and psoriasis share a similar clinical picture in terms of skin-specific symptoms. High EQ-5D index scores indicate good health, whereas low scores indicate poor health. For EQ-5D index, the MCID has been suggested to be at least 0.09 points in psoriasis patients (18). The total MADRS-S is based on 9 items, assessing “mood,” “appetite,” “zest for life,” “emotional involvement,” “ability to concentrate,” “initiative,” “pessimism,” “feelings of unease,” and “sleep.” The total MADRS-S score ranges from 0 to 54, with higher scores indicating more severe depressive symptoms. For VAS-itch, the MCID has been suggested to be at least 2.0 points (19).

In addition to patient-reported data, clinical data was collected and included CTCL subtype, clinical stage for MF/SS patients, mSWAT for MF/SS patients and present treatment for CTCL.

### 2.3 Statistical analysis

Fisher’s exact test and the chi-square test were used for comparisons between categorical variables. The Mann-Whitney test and the Kruskal-Wallis test with Dunn’s multiple comparisons test were used for comparisons between continuous variables, and Spearman’s rank correlation (Spearman’s ρ) was used for correlation analyses. P ≤ 0.05 was considered statistically significant. Values are given as median with interquartile range (IQR) and mean with range. All statistical analyses were performed using GraphPad Prism (version 9.2.0) and Microsoft Excel.

## 3 Results

### 3.1 Patient characteristics

Among the 76 CTCL patients included in the study, 64% were male. Sixty-one were diagnosed with MF or SS, 10 with LyP, 2 with PC-ALCL, and 3 with primary cutaneous peripheral T-cell lymphoma unspecified (**Table 1**). A majority of the 60 MF/SS patients with available data on stage had early-stage disease [stages IA–IIA: n = 58 (97%)] and low mSWAT (median 7, range 0–70). In the early-stage MF group, 34/58 (59%) presented with patches only and 24/58 (41%) presented with plaques with or without patches (**Supplementary Table 1**). There were no significant differences between male and female MF/SS patients in the median mSWAT, in the distribution of the different clinical stages, or in lesion type (data not shown). Most patients had no or topical treatment (88%) and the median time with CTCL diagnosis was 3 years.

**Table 1:** Patient characteristics of study cohort.

	Total sample	MF and SS	Other CTCLs	P^a^
Total (row%)	76 (100)	61 (80)	15 (19)	
Sex, n (%)				
Male	49 (64)	40 (66)	9 (60)	0.7665^b^
Female	27 (36)	21 (34)	6 (40)	
Age in years, median (range)	68 (27-90)	69 (27-90)	60 (32-79)	0.0955^c^
CTCL subtype, n (%)				
MF, no subtype	52 (68)	52 (85)		
Folliculotropic MF	8 (11)	8 (13)		
SS	1 (1)	1 (2)		
LyP	10 (13)		10 (67)	
ALCL	2 (3)		2 (13)	
Other	3 (39)		3 (20)	
Clinical stage, n (%)^d^				
IA		37 (61)		
IB		19 (31)		
IIA-IIIB		4 (7)		
mSWAT, median (range)^d^		7 (0-70)		
Treatment at hospital visit, n (%)				
None	16 (21)	10 (16)	6 (40)	0.0965^e^
Topical	51 (67)	42 (69)	9 (60)	
Systemic	9 (12)	9 (15)	0	
Disease duration				
Years from diagnosis, median (range)				
Median, range	3 (0-50)	3 (0-50)	3 (0-15)	0.6051^c^
≤ 2 years, n (%)	35 (46)	29 (48)	6 (40)	0.7738^b^
> 2 years, n (%)	41 (54)	32 (52)	9 (60)	
Years from symptom debut, median (range)^f^	11.5 (0-50)	12 (0-50)	8 (1-21)	**0.0369** ^c^

ALCL, cutaneous anaplastic large cell lymphoma; CTCL, cutaneous T cell lymphoma; LyP, lymphomatoid papulosis; MF, mycosis fungoides; mSWAT, modified Severity-Weighted Assessment Tool; SS, Sézary syndrome. Significant p-values highlighted in bold.

^a^For MF and SS vs other CTCLs.

^b^Fisher’s exact test.

^c^Mann-Whitney test.

^d^Data missing for 1 patient with MF.

^e^Fisher’s exact test for no treatment vs topical treatment.

^f^Data missing for 2 patients.

### 3.2 Overall small effects on QoL measures in the total CTCL patient group

In the total CTCL study group, there were generally only small effects reported on QoL, depression scores, and itch (**Table 2**). Based on the total DLQI scores, 61 (80%) of the CTCL patients had no to mild effect (0-5 points) on QoL, 3 (4%) had moderate effect (6-10 points) and 12 (16%) had very large to extremely large effect (11-30). Among the 71 patients who answered the MADRS-S questionnaire, 62 (87%) had no or very mild depression (0–12 points), 4 (6%) mild depression (13–19 points) and 5 (7%) moderate depression (20–34 points).

**Table 2:** Median and mean values of health-related quality of life (QoL) parameters, MADRS-S, and VAS-itch in CTCL patients (n=76), divided by sex.

	Total sample	Females	Males	P^a^
DLQI				
Median (IQR)	2.00 (1.00–4.75)	4.00 (1.00–7.00)	1.00 (0.50–4.00)	0.085
Mean (range)	4.47 (0.00–29.00)	5.74 (0.00–29.00)	3.78 (0.00–22.00)	
EQ-5D index				
Median (IQR)	0.80 (0.73–1.00)	0.73 (0.66–0.85)	0.85 (0.73–1.00)	**0.040**
Mean (range)	0.78 (-0.18–1.00)	0.72 (-0.18–1.00)	0.81 (-0.18–1.00)	
MADRS-S^b^				
Median (IQR)	4.00 (1.00–8.00)	4.00 (3.00–9.50)	4.00 (0.00–7.00)	0.088
Mean (range)	5.96 (0.00–29.00)	7.54 (0.00–29.00)	5.04 (0.00–27.00)	
VAS-itch				
Median (IQR)	0.90 (0.20–3.53)	1.05 (0.11–5.26)	0.63 (0.20–3.37)	0.435
Mean (range)	2.30 (0.00–10.00)	2.47 (0.00–10.00)	2.21 (0.00–10.00)	

DLQI, Dermatology Life Quality Index; EQ-5D, EuroQOL 5D; IQR, interquartile range; MADRS-S, Montgomery-Åsberg Depression Rating Scale – Self report; VAS, visual analogue scale. For the rest of the abbreviations, see **Table 1**. Statistically and clinically significant differences highlighted in bold.

^a^Mann-Whitney test.

^b^Data missing for 5 patients: 1 female and 4 males.

Female patients had significantly worse median EQ-5D index compared to male patients, p=0.040. No sex differences were observed for other primary outcomes, but the DLQI subscale “daily activities” and the MADRS-S item “sleep” were significantly impaired in females vs males (**Supplementary Table 2**). There were no differences of clear clinical significance in QoL measures between CTCL patients receiving no treatment, topical treatment and systemic treatment or between CTCL patients with a disease duration of more than 2 years and those with a duration of less than 2 years (**Supplementary Table 3** and data not shown).

### 3.3 Impaired QoL measures in MF/SS patients with higher disease activity and in female patients

Among the 61 patients with MF/SS and the subgroup of 58 MF patients with early stages IA-IIA, there were generally only small effects reported on QoL, depression scores and itch (**Table 3**). Females in the MF/SS-group, had significantly worse median EQ-5D index score compared to males, but the difference was not clinically significant in early-stage MF patients. Furthermore, females with MF/SS as well as early MF had significantly impaired DLQI subscale “daily activities” and the MADRS-S item “sleep” compared to males (**Supplementary Table 4**).

**Table 3:** Median and mean values of QoL parameters, MADRS-S, and VAS-itch in MF and SS patients (n = 61) divided by sex, including presentation of outcomes in early-stage IA-IIA disease (n = 58).

	Total sample	Females	Males	P^a^
DLQI				
Median (IQR)	2.00 (1.00–5.00)	4.00 (1.00–6.50)	2.00 (1.00–4.00)	0.0604
Mean (range)	4.67 (0.00–29.00)	5.64 (0.00–29.00)	4.07 (0.00–22.00)	
Analysis of early stages IA–IIA				
Median (IQR)	2.00 (1.00–5.00)	4.00 (1.00–7.00)	2.00 (1.00–4.00)	0.0361
Mean (range)	4.50 (0.00–29.00)	5.77 (0.00–29.00)	3.79 (0.00–22.00)	
EQ-5D index				
Median (IQR)	0.796 (0.725–1.000)	0.727 (0.691–0.848)	0.848 (0.726–1.000)	**0.0491**
Mean (range)	0.772 (-0.181–1.000)	0.715 (-0.181–1.000)	0.802 (-0.181–1.000)	
Analysis of early stages IA–IIA				
Median (IQR)	0.796 (0.725–1.000)	0.761 (0.673–0.848)	0.848 (0.727–1.000)	0.0417
Mean (range)	0.782 (-0.181–1.000)	0.715 (-0.181–1.000)	0.817 (-0.181–1.000)	
MADRS^b^				
Median (IQR)	4.00 (1.00–8.00)	4.00 (3.00–8.75)	4.00 (0.00–7.75)	0.1762
Mean (range)	5.89 (0.00–29.00)	7.10 (0.00–29.00)	5.22 (0.00–27.00)	
Analysis of early stages IA–IIA				
Median (IQR)	4.00 (1.00-8.00)	4.00 (3.00–9.00)	3.00 (0.00–7.00)	0.1071
Mean (range)	5.81 (0.00–29.00)	7.26 (0.00–29.00)	5.00 (0.00–27.00)	
VAS-itch				
Median (IQR)	1.05 (0.21–3.69)	1.16 (0.32–5.48)	0.84 (0.21–3.48)	0.6316
Mean (range)	2.46 (0.00–10.00)	2.67 (0.00–10.00)	2.36 (0.00–10.00)	
Analysis of early stages IA–IIA				
Median (IQR)	1.05 (0.21–3.63)	1.11 (0.32–5.59)	0.84 (0.21–3.27)	0.6111
Mean (range)	2.36 (0.00–10.00)	2.67 (0.00–6.45)	2.20 (0.00–10.00)	

For abbreviations, see **Tables 1**, **2**. Statistically and clinically significant differences highlighted in bold.

^a^Mann-Whitney test.

^b^Data missing for 5 patients: 1 female and 4 males.

In the total MF/SS population, as well as in the early MF subgroup, there was a weak positive correlation between mSWAT and DLQI as well as a weak negative correlation between mSWAT and EQ-5D index (**Table 4**). Furthermore, there were weak positive correlations between mSWAT and the DLQI subscales “symptoms and feelings” and “daily activities” as well as moderate positive correlation between mSWAT and the DLQI subscale “treatment” (**Supplementary Table 5**). A weak positive correlation was observed between VAS-itch and mSWAT in the total MF/SS group but not in the early MF group. There were no correlations between mSWAT and MADRS-S total scores, for MADRS-S items see **Supplementary Table 5**.

**Table 4:** Correlation analysis between mSWAT and QoL parameters, MADRS-S and VAS itch in MF/SS patients (n=60) as well as in early-stage IA-IIA disease (n=57).

	MF/SS		Early MF-stages IA-IIA	
	Spearman r	p	Spearman r	p
**DLQI**	0.4298	**0.0006**	0.4130	**0.0014**
**EQ-5D index**	-0.3557	**0.0053**	-0.3174	**0.0161**
**MADRS-S^a^**	0.0679	0.6189	0.0201	0.8867
**VAS-itch**	0.3221	**0.0121**	0.2849	0.0317

For abbreviations, see **Tables 1**, **2**. Significant p-values highlighted in bold.

^a^Data missing for 4 early-stage MF patients.

### 3.4 Impaired QoL and itch in early MF patients at stage IB and with plaque lesions

VAS-itch was increased in early-stage MF patients at stage IB, (patch/plaque covering 10-79% of BSA), compared to stage IA, (patch/plaque covering <10% of BSA), and QoL was impaired, as indicated by both an increase in DLQI and a decrease in EQ-5D index (**Table 5**). When analyzing the DLQI subscales, we observed a small but significant impairment in the “symptoms and feelings”, “daily activities” and “treatment” scores in MF patients at stage IB compared to patients at stage IA (**Supplementary Table 6**). The median total MADRS-S score was not significantly different between MF patients at stage IA and stage IB, for MADRS-S items, see **Supplementary Table 6**. The scores on the DLQI subscales and the MADRS-S items were generally low, and the median values in the groups were never more than 2 units.

**Table 5:** Median values of QoL parameters, MADRS-S, and VAS-itch in MF and SS patients divided by stage (n=56) and lesion type (n = 58).

	IA	IB	P^a^	Patch	Plaque	P^a^
DLQI						
Median (IQR)	1.00 (0.00–3.50)	5.00 (3.00–12.00)	**0.0006**	1.00 (0.00–3.25)	4.50 (2.00–12.00)	<0.0001
Mean (range)	2.60 (0.00–17.00)	7.95 (0.00–29.00)		2.09 (0.00–15.00)	7.92 (1.00–29.00)	
EQ-5D index						
Median (IQR)	0.848 (0.796–1.000)	0.725 (0.516–0.850)	**0.0040**	0.848 (0.796–1.000)	0.725 (0.491–0.849)	**0.0032**
Mean (range)	0.852 (0.331–1.000)	0.638 (-0.181–1.000)		0.867 (0.656–1.000)	0.661 (-0.181–1.000)	
MADRS-S^b^						
Median (IQR)	4.00 (0.00–7.00)	4.00 (0.25–12.50)	0.6371	4.00 (1.00–7.75)	4.00 (1.00–10.50)	0.6658
Mean (range)	4.86 (0.00–27.00)	7.81 (0.00–29.00)		4.63 (0.00–16.00)	7.62 (0.00–29.00)	
VAS-itch						
Median (IQR)	0.54 (0.20–1.53)	3.37 (0.32–7.47)	**0.0487**	0.43 (0.07–1.32)	3.27 (0.76–7.02)	**0.0006**
Mean (range)	1.51 (0.00–10.00)	3.86 (0.00–10.00)		1.21 (0.00–7.57)	3.99 (0.00–10.00)	

For abbreviations, see **Tables 1**, **2**. Statistically and clinically significant differences highlighted in bold.

^a^Mann-Whitney test.

^b^Data missing for 5 patients: 2 stage IA, 3 stage IB, 2 patch, and 3 plaque patients.

VAS-itch was increased in early-stage MF patients with plaques compared to patients with patches only and QoL was impaired, as indicated by a decrease in EQ-5D index (**Table 5**). In line with this, DLQI tended to be higher in patients with plaques, but the difference between the groups reached only nearly clinically significant levels. Analysis of DLQI subscales showed significantly higher scores for “symptoms and feelings”, “daily activities”, “leisure”, “work and school” and “treatment” in MF patients with plaques compared to those with patches (**Supplementary Table 6**). There were no significant differences in total MADRS-S scores. For MADRS-S items, see **Supplementary Table 6**.

### 3.5 QoL measures correlate with blood lactate dehydrogenase, eosinophils and hemoglobin

Among the CTCL patients with available blood test results within 6 months from the hospital visit, 18/66 (27%) had elevated plasma lactate dehydrogenase (LDH) levels, 4/67 (6%) had elevated blood eosinophils, 6/71 (8%) had mild anemia and 7/69 (10%) had elevated plasma creatinine levels. There were weak positive correlations between plasma LDH and worse QoL as measured with DLQI (r = 0.370, p = 0.0022), between blood hemoglobin (Hb) and better QoL as measured with EQ-5D index (r = 0.382, p = 0.0010) as well as between blood eosinophils and VAS-itch (r = 0.309, p = 0.0109). There were no correlations between plasma creatinine and the primary outcome measures. In the MF/SS patients, no associations were found between mSWAT, lesion types (patch or plaque) or early stages (IA or IB) and plasma LDH, plasma creatinine, blood eosinophils and blood hemoglobin (data not shown).

## 4 Discussion

In our broad analysis of QoL measures in CTCL patients we somewhat surprisingly found that patients with early-stage MF reported relatively little impact on QoL, depressive symptoms, and itch compared with what has been found in other studies (8, 20–22). This may partly be a result of the centralized and highly subsidized healthcare system in Sweden, resulting in a nearly population-based study cohort that includes a relatively large fraction of patients with milder manifestations of early-MF. Furthermore, national treatment guidelines for CTCL patients were implemented in Sweden the same year this study was initiated. This possibly made treatment and care of CTCL patients in Sweden better, and subsequently improved QoL measures in our cohort. In addition, only few patients in our cohort had advanced stages of MF/SS or rare subtypes of CTCL which likely influenced the QoL measures in the total MF/SS and CTCL study populations.

We, however, identified subgroups of early-stage MF patients with impaired QoL. These included patients with plaque lesions, who reported significant impairment in EQ-5D index, most DLQI subscales and itch compared to patients with patches only. The impaired QoL in patients with plaques could be caused by more severe symptoms or longer and more complicated treatments since plaques are often more difficult to treat than patches. Patients at stage IB also reported more itch and impaired QoL compared to patients at stage IA, both as measured with the EQ-5D index and DLQI. This possibly reflects larger suffering from the more spread disease and/or the cumbersome local treatment of larger BSA. Furthermore, higher disease activity correlated with impaired QoL and itch in early MF patients. Collectively, these findings are novel and of high clinical relevance, as treatment decisions in early stages of MF are largely guided by patients’ subjective symptoms.

Itch can affect several QoL factors and can lead to sleep disturbance and depression (9). In line with our results, a recent study suggests that itch can be used as an overall QoL indicator (20). Several medical conditions, including renal failure, hyper-eosinophilia, and anemia (23), can cause itch, and hence indirectly affect QoL. In our total CTCL cohort, anemia and hyper-eosinophilia were found in only 8% and 6% of the patients respectively. Conditions were generally mild but might partly explain worse itch or QoL in these patients. We observed a weak positive correlation between blood eosinophils and itch, indicating a potential role of eosinophils in the mechanism of itch in CTCL. In line with this, skin infiltration of eosinophils has previously been correlated with the severity of itch in MF (24). We further observed a weak positive correlation between Hb and better QoL, but no association between Hb and itch. This is possibly attributable to lower level of fatigue in patients with higher Hb levels. Elevated LDH is an indirect marker for increased tumor burden and has been previously identified as an independent adverse prognostic factor for survival in CTCL (25). In our CTCL cohort, LDH levels correlated with worse QoL, which is in line with the previously reported association between elevated LDH and worse QoL as measured with Skindex-29 (9). We, however, found no associations between blood LDH, eosinophils, hemoglobin or creatinine levels and MF/SS stage, lesion type or disease activity which indicates that the associations between QoL and MF/SS stage/disease activity are independent from other key medical conditions that might cause itch.

In the total CTCL population as well as in MF/SS patients, QoL was significantly impaired in females compared to males. Our data thus confirm previous findings of worse QoL in female CTCL patients as measured by Skindex-16 (9, 26).

We speculated that QoL would be more severely affected in patients with longer disease duration (> 2 years), but surprisingly found no such association in our cohort. This could be due to the fact that the majority of patients with early MF have a stable disease or that the patients received treatment to relieve itch and other symptoms (22).

To conclude, CTCL patients included in this study were mainly early-stage MF patients who reported relatively little impact on QoL. The fact that many early-stage MF patients experienced a high QoL is an important message to newly diagnosed patients. However, we identified subgroups of early-stage MF patients who reported impaired QoL and more itch. These included patients with plaque vs patches only as well as patients with stage IB vs IA, who therefore may be in need for more intensive treatment, monitoring, and support. Furthermore, higher disease activity correlated with both impaired QoL and more itch. Future studies are needed to determine QoL, itch and depressive symptoms in patients with rare subtypes of CTCL, as well as the effect on QoL measures of different treatment modalities.

## Data Availability

The raw data supporting the conclusions of this article will be made available by the authors in anonymized form, upon reasonable request. Requests to access the datasets should be directed to hanna.brauner@ki.se.
