# Supplementary material for: Identification of subgroups of early-stage mycosis fungoides patients with increased itch and impaired quality of life
Source: Front Oncol. 2025 Feb 28;15:1524353. doi: 10.3389/fonc.2025.1524353 (PMC11906425; doi:10.3389/fonc.2025.1524353)
Supplement: Supplementary file 1 [file DataSheet1.docx]

Supplementary Material

**Supplementary Table 1.** Patient characteristics of early MF patients (stages IA-IIA)

| **Sex**, n (%) |  |
| --- | --- |
| Male | 38 (66) |
| Female | 20 (34) |
| **Age** (years), median (range) | 62 (22-88) |
| **MF subtype**, n (%) |  |
| No subtype | 51 (88) |
| Folliculotropic MF | 7 (12) |
| **Clinical stage**, n (%) |  |
| IA | 37 (64) |
| IB | 19 (33) |
| IIA | 2 (3) |
| **Lesion type**, n (%) |  |
| Patches | 34 (59) |
| Plaque | 24 (41) |
| **mSWAT**, median (range) | 4.5 (0-62) |
| **Treatment at hospital visit**, n (%) |  |
| None | 9 (16) |
| Topical | 41 (71) |
| Systemic | 8 (14) |
| **Disease duration** |  |
| Years from diagnosis, median (range) |  |
| Median, range | 3 (0-50) |
| ≤ 2 years, n (%) | 27 (47) |
| > 2 years, n (%) | 31 (53) |
| Years from symptom debut, median (range)^a^ | 12 (0-50) |

MF, mycosis fungoides; mSWAT, modified Severity-Weighted Assessment Tool.

^a^Data missing for 2 patients

**Supplementary Table 2.** Median scores on DLQI subscales and MADRS-S items in CTCL patients (n=76), divided by sex.

|  | **Total Sample** | **Females** | **Males** | **P**^a^ |
| --- | --- | --- | --- | --- |
|  | **Median (IQR)** | **Median (IQR)** | **Median (IQR)** |  |
| **DLQI subscale** |  |  |  |  |
| Symptoms and feelings | 1.00 (0.00-2.00) | 1.00 (1.00-2.00) | 1.00 (0.00-2.00) | 0.1610 |
| Daily activities | 0.00 (0.00-1.00) | 1.00 (0.00-2.00) | 0.00 (0.00-0.00) | **0.0175** |
| Leisure | 0.00 (0.00-0.00) | 0.00 (0.00-1.00) | 0.00 (0.00-0.00) | 0.3171 |
| Work and school | 0.00 (0.00-0.00) | 0.00 (0.00-0.00) | 0.00 (0.00-0.00) | 0.7192 |
| Personal relationships | 0.00 (0.00-0.00) | 0.00 (0.00-1.00) | 0.00 (0.00-0.00) | 0.3046 |
| Treatment | 0.00 (0.00-1.00) | 0.00 (0.00-1.00) | 0.00 (0.00-1.00) | 0.2784 |
| **MADRS item**^b^ |  |  |  |  |
| Mood | 0.00 (0.00-0.00) | 0.00 (0.00-0.00) | 0.00 (0.00-0.00) | 0.8652 |
| Feelings of unease | 0.00 (0.00-2.00) | 0.50 (0.00-2.00) | 0.00 (0.00-2.00) | 0.2812 |
| Sleep | 2.00 (0.00-2.00) | 2.00 (1.00-4.00) | 1.00 (0.00-2.00) | **0.0236** |
| Appetite | 0.00 (0.00-2.00) | 0.00 (0.00-1.00) | 0.00 (0.00-0.00) | 0.1005 |
| Ability to concentrate | 0.00 (0.00-1.00) | 0.50 (0.00-2.00) | 0.00 (0.00-1.00) | 0.0702 |
| Initiative | 0.00 (0.00-1.00) | 0.00 (0.00-1.25) | 0.00 (0.00-1.00) | 0.2058 |
| Emotional Involvement | 0.00 (0.00-0.00) | 0.00 (0.00-1.00) | 0.00 (0.00-0.00) | 0.3060 |
| Pessimism | 0.00 (0.00-1.00) | 0.00 (0.00-1.00) | 0.00 (0.00-1.00) | 0.6122 |
| Zest for Life | 0.00 (0.00-1.00) | 0.00 (0.00-1.00) | 0.00 (0.00-0.00) | 0.4608 |

CTCL, cutaneous T cell lymphoma; DLQI, Dermatology Life Quality Index; IQR, interquartile range; MADRS-S, Montgomery-Åsberg Depression Rating Scale – Self report. Significant p-values highlighted in bold.

^a^Mann-Whitney test.

^b^Data missing for 5 patients: 1 female and 4 males.

**Supplementary Table 3.** Median values of health-related quality of life (QoL) parameters, MADRS-S, and VAS-itch in CTCL patients (n=76), divided by treatment.

|  | **None** | **Topical** | **Systemic** | **P^a^** |
| --- | --- | --- | --- | --- |
| **DLQI** |  |  |  |  |
| Median (IQR) | 1.00 (0.00-1.75) | 2.00 (1.00-5.00) | 4.00 (2.00-7.00) | 0.0326^c^ |
| Mean (range) | 2.38 (0.00-21.00) | 4.75 (0.00-22.00) | 6.67 (0.00-29.00) |  |
| **EQ-5D index** |  |  |  |  |
| Median (IQR) | 1.000 (0.809-1.000) | 0.796 (0.656-1.000) | 0.796 (0.725-0.796) | 0.0368^d^ |
| Mean (range) | 0.851 (-0.181-1.000) | 0.760 (-0.181-1.000) | 0.751 (0.516-0.848) |  |
| **MADRS-S^b^** |  |  |  |  |
| Median (IQR) | 4.00 (0.00-7.75) | 4.00 (1.25-7.00) | 11 (2.00-12.00) | 0.2432 |
| Mean (range) | 5.56 (0.00-22.00) | 5.44 (0.00-27.00) | 10.43 (0.00-29.00) |  |
| **VAS-itch** |  |  |  |  |
| Median (IQR) | 0.21 (0.00-0.69) | 1.29 (0.32-5.05) | 1.37 (0.15-5.11) | 0.0522 |
| Mean (range) | 1.15 (0.00-10.00) | 2.65 (0.00-10.00) | 2.38 (0.00-7.52) |  |

EQ-5D, EuroQOL 5D; VAS, visual analogue scale. For the rest of the abbreviations, see Supplementary Table 2.

^a^Kruskal Wallis test

^b^Data missing for 5 patients: 3 with topical and 2 with systemic treatment.

^c^Statistically but not clinically significant.

^d^Statistically significant with Kruskal Wallis test but not with Dunn’s multiple comparisons test (post-hoc test).

**Supplementary Table 4.** Median scores on DLQI subscales and MADRS-S items in MF and SS patients (n=61) divided by sex, including presentation of outcomes in early-stages IA-IIA disease (n=58).

|  | **Total sample** | **Females** | **Males** | **P**^a^ |
| --- | --- | --- | --- | --- |
|  | **Median (IQR)** | **Median (IQR)** | **Median (IQR)** |  |
| **DLQI subscale** |  |  |  |  |
| Symptoms and feelings | 1.00 (0.00-2.00) | 2.00 (1.00-2.00) | 1.00 (0.00-2.00) | 0.1800 |
| MF stages IA–IIA | 1.00 (0.00-2.00) | 2.00 (1.00-2.00) | 1.00 (0.00-2.00) | 0.1199 |
| Daily activities | 0.00 (0.00-1.00) | 1.00 (0.00-2.00) | 0.00 (0.00-0.00) | **0.0360** |
| MF stages IA–IIA | 0.00 (0.00-1.00) | 1.00 (0.00-2.00) | 0.00 (0.00-0.00) | **0.0156** |
| Leisure | 0.00 (0.00-0.00) | 0.00 (0.00-1.50) | 0.00 (0.00-0.00) | 0.4293 |
| MF stages IA–IIA | 0.00 (0.00-0.00) | 0.00 (0.00-1.75) | 0.00 (0.00-0.00) | 0.2680 |
| Work and school | 0.00 (0.00-0.00) | 0.00 (0.00-0.00) | 0.00 (0.00-0.00) | 0.7729 |
| MF stages IA–IIA | 0.00 (0.00-0.00) | 0.00 (0.00-0.00) | 0.00 (0.00-0.00) | 0.7720 |
| Personal relationships | 0.00 (0.00-0.00) | 0.00 (0.00-1.00) | 0.00 (0.00-0.00) | 0.4750 |
| MF stages IA–IIA | 0.00 (0.00-0.00) | 0.00 (0.00-1.00) | 0.00 (0.00-0.00) | 0.3360 |
| Treatment | 0.00 (0.00-1.00) | 0.00 (0.00-1.00) | 0.00 (0.00-1.00) | 0.4115 |
| MF stages IA–IIA | 0.00 (0.00-1.00) | 0.00 (0.00-1.00) | 0.00 (0.00-1.00) | 0.5549 |
| **MADRS item**^b^ |  |  |  |  |
| Mood | 0.00 (0.00-0.00) | 0.00 (0.00-0.00) | 0.00 (0.00-0.00) | 0.5234 |
| MF stages IA–IIA | 0.00 (0.00-0.00) | 0.00 (0.00-0.00) | 0.00 (0.00-0.00) | 0.5958 |
| Feelings of unease | 0.00 (0.00-2.00) | 0.00 (0.00-2.00) | 0.00 (0.00-1.75) | 0.4872 |
| MF stages IA–IIA | 0.00 (0.00-2.00) | 0.00 (0.00-2.00) | 0.00 (0.00-2.00) | 0.4986 |
| Sleep | 2.00 (0.25-3.75) | 2.00 (1.00-4.00) | 1.00 (0.00-2.00) | **0.0150** |
| MF stages IA–IIA | 1.00 (0.00-3.00) | 2.00 (1.00-4.00) | 1.00 (0.00-2.00) | **0.0163** |
| Appetite | 0.00 (0.00-0.00) | 0.00 (0.00-0.75) | 0.00 (0.00-0.00) | 0.2316 |
| MF stages IA–IIA | 0.00 (0.00-0.00) | 0.00 (0.00-1.00) | 0.00 (0.00-0.00) | 0.2299 |
| Ability to concentrate | 0.00 (0.00-1.00) | 0.00 (0.00-1.75) | 0.00 (0.00-1.00) | 0.4204 |
| MF stages IA–IIA | 0.00 (0.00-1.00) | 0.00 (0.00-2.00) | 0.00 (0.00-1.00) | 0.4367 |
| Initiative | 0.00 (0.00-1.00) | 0.00 (0.00-1.00) | 0.00 (0.00-1.00) | 0.4415 |
| MF stages IA–IIA | 0.00 (0.00-1.00) | 0.00 (0.00-1.00) | 0.00 (0.00-1.00) | 0.4348 |
| Emotional Involvement | 0.00 (0.00-0.00) | 0.00 (0.00-0.00) | 0.00 (0.00-0.00) | 0.8848 |
| MF stages IA–IIA | 0.00 (0.00-0.00) | 0.00 (0.00-0.00) | 0.00 (0.00-0.00) | 0.8839 |
| Pessimism | 0.00 (0.00-1.00) | 0.00 (0.00-1.00) | 0.00 (0.00-1.00) | 0.8645 |
| MF stages IA–IIA | 0.00 (0.00-1.00) | 0.00 (0.00-1.00) | 0.00 (0.00-1.00) | >0.9999 |
| Zest for Life | 0.00 (0.00-1.00) | 0.00 (0.00-0.75) | 0.00 (0.00-0.00) | >0.9999 |
| MF stages IA–IIA | 0.00 (0.00-1.00) | 0.00 (0.00-1.00) | 0.00 (0.00-0.00) | 0.8233 |

For abbreviations, see Supplementary Table 2. Significant p-values highlighted in bold.

^a^Mann-Whitney test.

^b^Data missing for 5 patients: 1 female and 4 males.

**Supplementary Table 5.** Correlation analysis between mSWAT and DLQI subscales and MADRS-S items in MF/SS patients (n=60) as well as early MF-patients (n=57).

|  | **MF/SS** | | **Early MF-stages IA-IIA** | |
| --- | --- | --- | --- | --- |
|  | **Spearman r** | **p** | **Spearman r** | **p** |
| **DLQI subscale** |  |  |  |  |
| Symptoms and feelings | 0.3766 | **0.0030** | 0.3437 | **0.0088** |
| Daily activities | 0.4122 | **0.0011** | 0.3952 | **0.0023** |
| Leisure | 0.2469 | 0.0572 | 0.2050 | 0.1262 |
| Work and school | 0.0977 | 0.4576 | 0.1176 | 0.3834 |
| Personal relationships | 0.1227 | 0.3504 | 0.0678 | 0.6166 |
| Treatment | 0.5336 | **<0.0001** | 0.5277 | **<0.0001** |
| **MADRS-S item^a^** |  |  |  |  |
| Mood | 0.4417 | **0.0007** | 0.4193 | **0.0018** |
| Feelings of unease | 0.0220 | 0.8720 | 0.0607 | 0.6660 |
| Sleep | 0.0071 | 0.9582 | -0.0583 | 0.6782 |
| Appetite | 0.0835 | 0.5407 | 0.0994 | 0.4788 |
| Ability to concentrate | -0.0144 | 0.9159 | 0.0214 | 0.8790 |
| Initiative | 0.2146 | 0.1123 | 0.2596 | 0.0605 |
| Emotional Involvement | 0.2678 | 0.0460 | 0.3047 | **0.0266** |
| Pessimism | 0.0833 | 0.5417 | 0.0560 | 0.6903 |
| Zest for Life | 0.2735 | 0.0414 | 0.2274 | 0.1015 |

mSWAT, modified Severity-Weighted Assessment Tool. For the rest of the abbreviations, see Supplementary Table 2. Significant p-values highlighted in bold.

^a^Data missing for 4 early-stage MF patients.

**Supplementary Table 6.** Median scores on DLQI subscales and MADRS-S items in MF and SS patients, divided by stage (n=56) and lesion type (n = 58).

|  | **IA** | **IB** | **P^a^** | **Patch** | **Plaque** | **P^a^** |
| --- | --- | --- | --- | --- | --- | --- |
|  | **median (IQR)** | **median (IQR)** |  | **Median (IQR)** | **Median (IQR)** |  |
| **DLQI subscale** |  |  |  |  |  |  |
| Symptoms and feelings | 1.00 (0.00–2.00) | 2.00 (1.00–3.00) | **0.0047** | 1.00 (0.00–2.00) | 2.00 (1.00–3.00) | **0.0015** |
| Daily activities | 0.00 (0.00–0.00) | 1.00 (0.00–4.00) | **0.0022** | 0.00 (0.00–0.25) | 0.50 (0.00–2.75) | **0.0112** |
| Leisure | 0.00 (0.00–0.00) | 0.00 (0.00–1.00) | 0.0766 | 0.00 (0.00–0.00) | 0.00 (0.00–2.00) | **0.0026** |
| Work and school | 0.00 (0.00–0.00) | 0.00 (0.00–1.00) | 0.1230 | 0.00 (0.00–0.00) | 0.00 (0.00–1.00) | **0.0372** |
| Personal relationships | 0.00 (0.00–0.00) | 0.00 (0.00–1.00) | 0.2542 | 0.00 (0.00–0.00) | 0.00 (0.00–1.75) | 0.0751 |
| Treatment | 0.00 (0.00–1.00) | 1.00 (0.00–1.00) | **0.0020** | 0.00 (0.00–0.00) | 1.00 (0.25–1.00) | **<0,0001** |
| **MADRS-S item^b^** |  |  |  |  |  |  |
| Mood | 0.00 (0.00–0.00) | 0.00 (0.00–1.25) | **0.0408** | 0.00 (0.00–0.00) | 0.00 (0.00–1.50) | 0.1004 |
| Feelings of unease | 0.00 (0.00–2.00) | 0.00 (0.00–1.25) | 0.6840 | 0.00 (0.00–2.00) | 0.00 (0.00–2.00) | 0.5451 |
| Sleep | 1.00 (0.00–2.50) | 1.00 (0.00–2.25) | 0.5859 | 2.00 (0.25–2.75) | 1.00 (0.00–4.00) | 0.8510 |
| Appetite | 0.00 (0.00–0.00) | 0.00 (0.00–1.00) | 0.1233 | 0.00 (0.00–0.00) | 0.00 (0.00–0.00) | 0.6320 |
| Ability to concentrate | 0.00 (0.00–1.00) | 0.00 (0.00–1.25) | 0.8327 | 0.00 (0.00–1.00) | 0.00 (0.00–1.50) | 0.3497 |
| Initiative | 0.00 (0.00–1.00) | 0.00 (0.00–2.00) | 0.1161 | 0.00 (0.00–1.00) | 0.00 (0.00–1.50) | 0.5924 |
| Emotional involvement | 0.00 (0.00–0.00) | 0.00 (0.00–1.00) | 0.3365 | 0.00 (0.00–0.00) | 0.00 (0.00–1.00) | **0.0406** |
| Pessimism | 0.00 (0.00–1.00) | 0.00 (0.00–1.00) | 0.7925 | 0.00 (0.00–0.75) | 0.00 (0.00–1.00) | 0.1319 |
| Zest for life | 0.00 (0.00–0.00) | 0.00 (0.00–1.00) | 0.2560 | 0.00 (0.00–0.00) | 0.00 (0.00–1.00) | 0.3834 |

For abbreviations, see Supplementary Table 2. Significant p-values highlighted in bold.

^a^Mann-Whitney test.

^b^Data missing for 5 patients.
